# Physicians’ Vacations

**Published:** 1879-09

**Authors:** James I. Tucker


### Article XVI.

### Physicians' Vacations.

*Editors of the* Medical Journal and Examiner :

What the *Northwestern Christian Advocate* says of ministerial vacations applies equally to physicians. The physician as well as the minister will come back from a sojourn in the country, the mountains or at the sea shore, "with fresh coals from off the altar, and such return will quicken his own zeal, and mutual good will follow." His people, as well as the minister's, all take a vacation, and are willing to give a vacation. It is undeniable that physicians, of all people, are most indifferent to their own health, and nothing but that happy division of labor into mental and physical, sedentary and out-door life, accounts for the good average health of the profession, in spite of constant, uninterrupted application to the most difficult and dangerous of arts. The legitimate medical work is not a lucrative one, as every physician knows, notwithstanding a good deal of boasting on the false principle that pretended success begets success, and the popular opinion. But there are few physicians who cannot lay aside a few hundred dollars for a quiet summer vacation with his family and friends. And as things go now, he will probably find wherever he may go among the popular places of resort, many of his families who will rejoice at his presence, and that they are not compelled to entrust themselves to strange physicians, who, destitute of that intimate knowledge with constitutions and temperaments which the family physician alone possesses, have no resource but the raw principles of their art. Some of the older physicians go away for the express purpose of emancipating themselves from the laboriousness of their professional work, but the young man, and many at middle age, dare not leave home, lest they suffer much loss and their hard-earned triumphs be neutralized. But if the experience of some of the physicians who have left home for health and recreation be considered, it will be seen that they have not only not lost but gained. My late partner in Boston was absent two years in Europe, Egypt and the Holy Land, and the very day of his return he had as many calls as he could attend to — not because he put forth a personal effort, but because his return had been waited for with expectancy, and *that succeeding year's work* repaid him in money, for his mind had been invigorated and enriched and his physical strength so much increased that he worked without friction. Then, too, we all recollect the experience of Sir Henry Holland, who, at the outset of his long and successful medical career laid out a scheme of annual travel. He says it was in no way injurious to him, but adds: "Had I not been attached to my profession, and had it not happened that my practice lay chiefly among the classes who are absent from London in the autumn, the result might have been different." His early resolution as to the matter of travel, steadily persevered in, proved gain to him in all succeeding life. "I have come back each year," says he, "refreshed in health of body and mind, and ready for the ten months of busy practice which lay before me. On the day or even hour of reaching home from long and distant journeys, I have generally resumed my wonted professional work. The new methods of inter-communication since steam and electricity have held empire upon the earth, often enabled me to make engagements for the very moment of my return." He took long journeys, and was absent a long time, and was known the world over, not only as one of the greatest of physicians, but one of the greatest of travelers. Thus he had the amplest opportunity to study *man*, which is always better than the study of books, especially to one jaded out with professional labor. It is not necessary, however, to take long journeys, or aspire to the reputation of a cosmopolite in travel, but for mental and bodily rest and invigoration to take ourselves that change of air, scene, diet and companionship which other professions find necessary, and which we all recommend to our patients.

DR. JAMES I. TUCKER.

